# Global Incidence of Carbapenemase-Producing *Escherichia coli* ST131

**DOI:** 10.3201/eid2011.141388

**Published:** 2014-11

**Authors:** Gisele Peirano, Patricia A. Bradford, Krystyna M. Kazmierczak, Robert E. Badal, Meredith Hackel, Daryl J. Hoban, Johann D.D. Pitout

**Affiliations:** University of Calgary Faculty of Medicine, Calgary, Alberta, Canada (G. Peirano, J.D.D. Pitout);; AstraZeneca Pharmaceuticals LP, Waltham, Massachusetts, USA (P.A. Bradford);; International Health Management Associates, Schaumburg, Illinois, USA (K.M. Kazmierczak, R.E. Badal, M. Hackel, D.J. Hoban)

**Keywords:** Antimicrobial drug resistance, antibiotic, antibacterial, surveillance, carbapenem, carbapenemases, *Escherichia coli*, ST131, bacteria, expedited

## Abstract

We characterized *Escherichia coli* ST131 isolates among 116 carbapenemase-producing strains. Of isolates from 16 countries collected during 2008–2013, 35% belonged to ST131 and were associated with *bla*_KPC_, *H*30 lineage, and virotype C. This study documents worldwide incidents of resistance to “last resort” antimicrobial drugs among a common pathogen in a successful sequence type.

*Escherichia coli* sequence type 131 (ST131) was identified as pathogenic to humans in 2008; retrospective research suggests that its isolates have been present since at least 2003. The group has spread extensively and has been linked to the rapid global increase in the prevalence of antimicrobial resistance among *E. coli* strains (*1*). The intercontinental dissemination of this sequence type has contributed immensely to the worldwide emergence of fluoroquinolone-resistant and CTX-M–producing *E. coli* (*1*,*2*). Recent surveillance studies have shown that its overall prevalence ranges from 12.5% to 30% of all *E. coli* clinical isolates, from 70% to 80% of fluoroquinolone-resistant isolates, and from 50% to 60% of extended spectrum beta-lactamase-producing isolates (*3*).

The development of resistance to carbapenems among *E. coli* is of particular concern because these agents are often the last line of effective therapy available for the treatment of persons with serious infections (*4*). New Delhi metallo-β-lactamase (NDM) and carbapenem-hydrolyzing oxacillinase-48 (OXA-48) are the most common carbapenemases among *E. coli* worldwide (*5*).

## The Study

This study describes the characteristics of ST131 isolates among carbapenemase-producing *E. coli* strains collected globally by 2 research groups during 2008–2013. The Merck Study for Monitoring Antimicrobial Resistance Trends **(**SMART) (http://www.merck.com/about/featured-stories/infectious_disease.html) started in 2002 and AstraZeneca's global surveillance study of antimicrobial resistance (unpublished data) began in 2012, to monitor global antimicrobial resistance trends among gram-negative bacteria (Technical Appendix). Antimicrobial susceptibilities of different antimicrobial agents (Table 1) were determined by using frozen broth microdilution panels according to 2013 Clinical and Laboratory Standards Institute and European Committee on Antimicrobial Susceptibility Testing guidelines (*6*). Established PCR and sequencing methods were used to identify β-lactamase genes (*7*,*8*) and define O25b:H4, O16:H5 ST131, *fimH*30 lineage, *H*30-Rx sublineage (*9*–*11*), and virotypes (*12*).

**Table 1:** Antimicrobial drug susceptibilities, β-lactamase types, *fimH*30 lineage, and different virotypes of *Escherichia coli* that have carbapenemases*

Characteristic	No. (%) non-ST131,n = 75	No. (%) ST131, n = 41	Total no. (%), n=116	p value
Antimicrobial drug nonsusceptible				
Ampicillin-sulbactam	75 (100)	41 (100)	116 (100)	1
Piperacillin-tazobactam	75 (100)	41 (100)	116 (100)	1
Ceftriaxone	65 (87)	39 (95)	104 (90)	0.5
Ceftazidime	65 (87)	39 (95)	104 (90)	0.5
Cefepime	65 (87)	39 (95)	104 (90)	0.5
Cefoxitin	65 (87)	39 (95)	104 (90)	0.5
Ertapenem	68 (91)	40 (98)	108 (93)	0.4
Imipenem	71 (95)	40 (98)	111 (96)	1
Amikacin	29 (39)	16 (39)	45 (39)	1
Ciprofloxacin	46 (61)	40 (95)	86 (74)	<0.001
Tigecycline	2 (3)	1 (2)	3 (3)	NA
Colistin	0	0	0	NA
Type of β-lactamase				
NDM	41 (55)	3 (7)	44 (38)	<0.001
KPC	14 (19)	24 (59)	38 (32)	<0.001
OXA-48-like	17 (23)	13 (32)	30 (26)	0.2
VIM	2 (3)	0	2 (2)	NA
IMP	1 (1)	1 (2)	2 (2)	NA
CTX-M-15	39 (52)	15 (37)	54 (47)	0.1
*H*30 lineage	35 (47)	24 (58)	59 (51)	0.3
Virotype				
A	1 (1)	0	1	NA
B	8 (11)	0	8	NA
C	9 (12)	39 (95)	48 (41)	<0.001
D	4 (5)	2 (5)	6 (5)	NA
ND	53 (71)	0	53 (46)	NA

*Nonsusceptible, either intermediate or resistant; NA, not available (too many cells numbering <5); ND: not detected; analysis was done using Stata version 10.0 (Stata Corp, College Station, TX, USA). χ^2^ and Fisher exact tests were used to compare group categorical data; NDM, New Delhi metallo-β-lactamase-1; KPC, *Klebsiella pneumoniae* carbapenemase; OXA, oxacillinase; VIM, Verona integron–encoded metallo-β-lactamase; IMP, imipenemase.

Overall, 47,843 *E. coli* isolates were collected and tested for susceptibility; 407 were found to be nonsusceptible to ertapenem or imipenem and were molecularly characterized for β-lactamase genes. A total of 116 of the 407 isolates were positive for NDM, KPC, OXA-48-like, VIM, and IMP types of carbapenemases. Various gene types were identified: 44 (38%) were positive for *bla*_NDM_, 38 (33%) for *bla*_KPC_, 30 (26%) for *bla*_OXA-48-like_, 2 (2%) for *bla*_VIM-1_ and 2 (2%) were positive for *bla*_IMP_ (Table 1).

The countries from which the *E. coli* isolates were obtained are shown in Table 2. The isolates were cultured from intraabdominal specimens (37%), peritoneal fluid (16%), biliary fluid (10%), urine (30%), and from miscellaneous sources such as sputum and tissue (9%).

**Table 2:** *Escherichia coli* with carbapenemases from combined Merck Study for Monitoring Antimicrobial Resistance Trends and AstraZeneca surveillance programs*

Carbapenemase (no.)	Total: country (no.)	ST131: country (no.)†
NDM (44)		
NDM-1 (39)	India (25), Vietnam (10), Serbia (1), Philippines (1), Thailand (1), China (1)	Philippines (1), India (1), Thailand (1)
NDM-4 (2)	India (2)	None
NDM-5 (n2)	Saudi Arabia (1), Kuwait (1)	None
NDM-6 (n1)	India (1)	None
KPC (38)		
KPC-2 (32)	Argentina (1), Brazil (2), Colombia (9), China (5), Ecuador (2), Italy (1), Jordan (1), Panama (1), Puerto Rico (5), USA (2), Vietnam (3)	Argentina (1), Colombia (5), China (4), Ecuador (1), Italy (1), Panama (1), Puerto Rico (4), USA (2), Vietnam (2)
KPC-3 (6)	Puerto Rico (1), Israel (1), USA (4)	USA (3)
OXA-48-like (30)		
OXA-48 (28)	Egypt (1), Jordan (1), Lebanon, (3), Morocco (2), Turkey (18), Vietnam (3), UAE (1)	Jordan (1), Morocco (1), Turkey (10), UAE(1)
OXA-163 (1)	Argentina (1)	None
OXA-244 (1)	Tunisia (1)	None
IMP (2)		None
IMP-1 (1)	India (1)	None
IMP-14 (1)	Thailand (1)	Thailand (1)
VIM-1 (2)	Italy (1), Greece (1)	None
Total	116	41

*NDM, New Delhi metallo-β-lactamase-1; KPC, *Klebsiella pneumoniae* carbapenemase; USA, United States of America; OXA, oxacillinase; UAE, United Arab Emirates; IMP, imipenemase; VIM, Verona integron–encoded metallo-β-lactamase. †PCR-based screening of *E. coli* ST131 may infrequently identify isolates that belong to the 131 Clonal Complex as ST131 and rarely misidentifies non-ST131 *E. coli* as ST131.

PCR testing for O25b:H4, O16:H5, and MLST showed that 41/116 (35%) belonged to the sequence type ST131. Antimicrobial susceptibilities, types of β-lactamases, the presence of the *fimH*30 lineage, and virotypes are shown in Table 1. ST131strains were more likely than non-ST131 strains to be nonsusceptible to ciprofloxacin and to be positive for *bla*_KPC_, the *H*30 lineage, and virotype C; non-ST131 isolates were more likely to be positive for *bla*_NDM_.

The majority, i.e., 24 (58%), of ST131strains were positive for *bla*_KPC_, 13 (32%) for *bla*_OXA-48-like_, 3 (7%) for *bla*_NDM-1_, and 1 (2%) for *bla*_IMP-14_. ST131 was present in 16 countries spanning 5 continents (Table 2). The distribution of ST131 during 2008–2013 is shown in Table 3.

**Table 3:** Temporal distribution of *Escherichia coli* ST131 in 2 global studies, 2008–2013*

Year	Total no. *E. coli*	No. carbapenem-nonsusceptible *E. coli*	No. (%) carbapenemase-producing *E. coli*	Type of carbapenemases (no.)	No. ST131	fim*H*30	Type of carbapenemases among ST131 (no.)
2008	3,739	45	10 (0.3)	NDM-1 (9), IMP-1 (1)	0	0	0
2009	5,913	63	21 (0.4)	NDM-1(16), NDM-4 (2), NDM-6 (1), OXA-48 (2)	1	0	NDM-1 (1)
2010†	8,951	71	17 (0.2)	KPC-2 (7), OXA-48 (10)	17	10	KPC-2 (7), OXA-48 (10)
2011	10,009	81	21 (0.2)	KPC-2 (9), KPC-3 (1), NDM-1 (5), OXA-48 (5), OXA-163 (1)	8	2	KPC-2 (6), KPC-3 (1), NDM-1 (1)
2012‡	14,275	97	35 (0.2)	KPC-2 (n12), KPC-3 (2), NDM-1 (7), NDM-5 (1), OXA-48 (11), OXA-244 (1), IMP-14 (1)	9	7	KPC-2 (5), OXA-48 (3), IMP-14 (1)
2013	4,956	50	12 (0.2)	KPC-2 (4), KPC-3 (3), NDM-1 (2), NDM-5 (1), VIM-1 (2)	6	5	KPC-2 (3), KPC-3 (2), NDM-1 (1)
Total	47,843	407	116	NDM-1 (39), NDM-4 (2), NDM-5 (2), NDM-6 (1), KPC-2 (32), KPC-3 (6), OXA-48 (28), OXA-163 (1), OXA-244 (1), IMP-1 (1), IMP-14 (1), VIM-1 (2)	41	24	KPC-2 (21), KPC-3 (3), NDM-1 (3), OXA-48 (13), IMP-14 (1)

*The 2 studies were the Merck Study for Monitoring Antimicrobial Resistance Trends** (**SMART) and the AstraZeneca global antimicrobial drug surveillance program. Isolates from SMART were not available for analysis in 2013: during 2008–2009, 1/32 (3%) *E. coli* isolates with carbapenemases from SMART were ST131 as opposed to 13/44 (30%) during 2011–2012. The limitation of the current study is that it uses a convenience set of isolates and differences over time could be related to differences in sampling rather than true increases in prevalence. Isolates from India were only obtained during 2008–10 while isolates from China were submitted in 2008, 2012 and 2013. NDM, New Delhi metallo-β-lactamase-1; IMP, imipenemase; OXA, oxacillinase, KPC, Klebsiella pneumonia carbapenemase; VIM, Verona integron–encoded metallo-β-lactamase. †ST131 from 2010 should be interpreted with caution because 9 of the 17 isolates were submitted from a single hospital within Turkey. These isolates were positive for *bla*_OXA-48_, *bla*_CTXM-15_, and belonged to the *H*30-R sublineage. It is likely that this institution housed an outbreak during that time. If the 2010 isolates are removed from consideration, there was a substantial increase in ST131 toward the latter part of this study. ‡The AstraZeneca global surveillance program was initiated in 2012.

Various *fimH* alleles were identified among ST131 isolates: 24 *H*30 (58%), 3 H41 (7%), 3 *H*54 (7%), 2 *H*22 (5%), 2 *H*27 (5%), and 2 *H*191 (5%); and 1 each (2%) belonging to *H*24, *H*32, *H*65, and the new fim*H* alleles *H*434 and *H*435. Of the 24 *H*30 ST131 strains, 19 (79%) belonged to the *H*30-R sublineage and 5(21%) to the *H*30-Rx sublineage.

## Conclusions

NDM variants were the most common carbapenemase identified and were especially prevalent in *E. coli* strains from India and Vietnam (Table 2). KPCs, which were the second most common carbapenemase identified, were distributed globally, i.e., in South America, Central America, North America, Europe, the Middle East, and Asia (Table 2). This was unexpected because KPCs have been relatively rarely reported among *E. coli* (*5*).

Because of the unprecedented global success of ST131, the presence of carbapenemases had been carefully monitored by molecular epidemiologists but has been limited to case reports from several countries (*1*). The largest collections of ST131 with carbapenemases were reported from Hangzhou, Zhejiang Province, China (*13*) and Pittsburgh, Pennsylvania, USA (*14*). Of note, 24/38 (63%) of *E. coli* strains with *bla*_KPC_ belonged to ST131, as opposed to 3/44 (7%) for NDMs and 13/30 (43%) for OXA-48-like strains. Our results suggest that ST131 is most likely responsible for the global distribution of *E. coli* with *bla*_KPC._

The expansion of the *H*30 lineage and its *H*30-R and *H*30-Rx sublineages have contributed substantially to the spread of ST131 *E. coli* (*11*,*15*). In our study, *H*30-R, which belongs to virotype C, was the most common lineage among ST131 strains (i.e., 58%); it was associated with *bla*_KPC_ and was especially prominent during 2012–2013. The increase of the ST131 *H*30 lineage with *bla*_KPC_ during 2012–13 is cause for concern.

*E. coli* ST131 has received comparatively less attention than other antimicrobial-resistant pathogens. Retrospective molecular surveillance studies have shown that ST131 with *bla*_CTX-M-15_ was rare during the early 2000s, but that an explosive global increase followed during the mid-to-late 2000s (*1*). The results of this study show a similar scenario with *E. coli* ST131 and *bla*_KPC_; a low prevalence combined with a global distribution. This study is of special concern because we documented resistance to “last resort” antimicrobial drugs (i.e., carbapenems) in most regions of the world, in a common community and hospital pathogen (i.e., *E. coli*) among a very successful sequence type (i.e., ST131). We urgently need well-designed epidemiologic and molecular studies to clarify the dynamics of transmission, risk factors, and reservoirs for ST131.

The medical community can ill afford to ignore *E. coli* ST131strains with carbapenemases. This sequence type poses a major threat to public health because of its worldwide distribution and association with the dominant *H*30 lineage. This sequence type among *E. coli* has the potential to cause widespread resistance to carbapenems.

Technical Appendix A brief description is provided of antimicrobial resistance surveillance and molecular characterization programs by pharmaceutical companies Merck and AstraZeneca.
