# Supplementary material for: Global Incidence of Carbapenemase-Producing Escherichia coli ST131
Source: Emerg Infect Dis. 2014 Nov;20(11):1928–31. doi: 10.3201/eid2011.141388 (PMC4214325; doi:10.3201/eid2011.141388)
Supplement: Technical Appendix — A brief description is provided of antimicrobial resistance surveillance and molecular characterization programs by pharmaceutical companies Merck and AstraZeneca. [file 14-1388-Techapp-s1.pdf]

DOI: http://dx.doi.org/10.3201/eid2011.141388

Global Incidence of Carbapenemase-Producing *Escherichia coli* ST131

The Studies

The Merck Study for Monitoring Antimicrobial Resistance Trends (SMART) and AstraZeneca program antimicrobial drug surveillance program combine global surveillance with molecular characterization to monitor the prevalence of different resistance determinants and sequence types among Gram negative bacteria. The SMART surveillance program included 237 institutions from 56 countries and the AstraZeneca surveillance program included 180 institutions from 39 countries present in Africa (5%), Asia (25%), Latin America (27%), Europe (13%), North America (17%), the South Pacific (5%) and the Middle East (8%). Countries that are represented include Argentina, Australia, Brazil, Canada, China, Chili, Colombia, Dominican Republic, Ecuador, Egypt, Greece, Costa Rica, Czech Republic, Estonia, France, Germany, Guatemala, Israel, India, Italy, Japan, Jordan, Honduras, Hong Kong, Korea, Kuwait, Lebanon, Latvia, Lithuania, Mexico, Morocco, New Zealand, Panama, Poland, Oman, the Philippines, Paraguay, Portugal, Puerto Rico, Romania, Saudi Arabia, Serbia, Singapore, Slovenia, Slovakia, Spain, Switzerland, Taiwan, Thailand, Turkey, Tunisia, United Arab Emirates, United Kingdom, Uruguay, USA, Venezuela, and Vietnam.

Each investigator from both programs was instructed to collect 100 consecutive clinically relevant Gram-negative aerobes from intra-abdominal infections and 50 from urinary tract infections. All isolates that were deemed to be colonizers were not collected. Astra-Zeneca specifically targeted *E. coli*, *Klebsiella* spp., *Proteus* spp., *Enterobacter* spp., *Citrobacter* spp., and *Serratia* spp. and also collected gram-negative bacteria responsible for skin and soft tissue infections and lower respiratory tract infections.

The bacteria with carbapenemases were collected during 2008–2013. Of 47,843 *E. coli* isolates, 4% were from Africa, 28% from Asia, 32% from Latin America, 9% from Europe, 14% from North America, 5% from the South Pacific and 8% the Middle East (8%).
